# High-Accuracy Correction of a Microlens Array for Plenoptic Imaging Sensors

**DOI:** 10.3390/s19183922

**Published:** 2019-09-11

**Authors:** Suning Li, Yuan Yuan, Ziyi Gao, Heping Tan

**Affiliations:** Key Laboratory of Aerospace Thermophysics, Ministry of Industry and Information Technology, School of Energy Science and Engineering, Harbin Institute of Technology, Harbin 150001, China; 16B902036@stu.hit.edu.cn (S.L.); 18S002009@stu.hit.edu.cn (Z.G.); tanheping@hit.edu.cn (H.T.)

**Keywords:** plenoptic camera, microlens array, error correction, light-field image processing

## Abstract

Microlens array (MLA) errors in plenoptic cameras can cause the confusion or mismatching of 4D spatio-angular information in the image space, significantly affecting the accuracy and efficiency of target reconstruction. In this paper, we present a high-accuracy correction method for light fields distorted by MLA errors. Subpixel feature points are extracted from the microlens subimages of a raw image to obtain correction matrices and perform registration of the corresponding subimages at a subpixel level. The proposed method is applied for correcting MLA errors of two different categories in light-field images, namely form errors and orientation errors. Experimental results show that the proposed method can rectify the geometric and intensity distortions of raw images accurately and improve the quality of light-field refocusing. Qualitative and quantitative comparisons between images before and after correction verify the performance of our method in terms of accuracy, stability, and adaptability.

## 1. Introduction

Plenoptic imaging is an advanced computational photography technique that can obtain both spatial and angular information of light rays in a single exposure [1,2]. The retention of radiation information in the angular dimension provides the necessary light-field data for altering the viewpoints or focusing depth of the target scene, which enables the capabilities of depth estimation, dynamic capture, and volumetric reconstruction [2,3,4,5]. This means that plenoptic imaging systems can be used as sensors for making measurements in complex scenarios, such as high-temperature flames [6,7,8], 3D fluid flow fields [9,10], and organ microtopography [11]. In such systems, a microlens array (MLA) built in front of the image sensor plays an important role in recording 4D light field. It disperses the light rays from the main lens onto the sensor pixels to form a series of microlens subimages that constitute a light field raw image. Each subimage holds 2D spatial information, and the sequential pixels beneath it contain 2D angular information. Owing to this correlation, the accuracy of light-field data is affected by the MLA parameters. To support the use of plenoptic imaging sensor in measurement applications, MLAs must maintain a predesigned geometric form and remain aligned with internal components. Similarly, in artificial compound-eye imaging sensors and other stretchable optoelectronic imaging systems [12,13], the use of improved MLA structures such as elastomeric MLAs and curved MLAs, opens up the potential for wide-angle field of view and infinite depth of field. The geometric parameters of the built-in MLAs also determine the optical performance of these imaging systems.

However, variability in MLAs is inevitably introduced in the manufacturing and assembly stages, and thus the captured raw images have geometric and intensity distortions. For example, displacements and aliasing artifacts occur among subimages owing to the orientation errors between the MLA and sensor planes [14,15,16], and there are the deviations in the brightness, clarity, and projection center of the subimages caused by microlens form errors [17,18]. Extant researches shows that these distortions deteriorate the quality of light-field reconstruction. In particular, confusion or loss of data results in nonlinear distortion of light-field refocused images [16,17,19], which in turn, leads to inaccurate measurement results [20,21,22,23]. In recent work, researchers have attempted to calibrate the MLA errors in plenoptic imaging systems and correct the resulting distortions in raw images. Suliga et al. [24] proposed a calibration method for MLAs in unfocused plenoptic cameras where the centers of the microlens subimages are estimated using a single image to determine the position offsets of the MLA with relation to the image sensor. Su et al. [25] formulated a geometric projection model that considers the translation and rotation errors of the MLA and applied the obtained error functions in optimization algorithms for the MLA orientation parameters. Bok et al. [26] presented a method for the geometric calibration of plenoptic cameras. The extrinsic parameters of the MLA are estimated and optimized using line features extracted from raw images. However, large errors remain at outer regions of the images after this calibration procedure because certain aberrations and errors from the microlenses are neglected in their model. Moreover, Cho et al. [27] attempted to deal with the MLA rotation errors for commercial Lytro cameras and correct the image distortion based on parameter calibration. Jin et al. [28] employed a similar technique to estimate MLA orientation errors via the fitting of subimage centers and by adjusting the entire raw image to the horizontal axis for rectifying the distorted light field.

The current approaches for MLA error calibration and image correction generally focus on the orientational offsets between the array and the image sensor while neglecting form errors based on the simplification that the microlens is considered as a pinhole model or a thin-lens model. Thus, there are some limitations to these simplified models and methods. First, parameter calibration algorithms depend on the estimated subimage centers. However, integer pixel levels are not adequate for locating the exact center points, causing variation in the solved parameters from the ground truths. Second, microlens errors and various aberrations of the main lens may affect the shape and uniformity of the subimages, which also aggravates the inaccuracy of parameter estimation and projection error corrections. Third, the types and values of the form errors that occur on each microlens are distinct, and even the degradation of subimages owing to the same source of error can be different [18] such that integral correction techniques are neither effective nor efficient for local microlens errors and their corresponding distortions.

In the previous work [29], a method was proposed for rectifying the image distortions caused by MLA form errors. However, it yields an undesirable effect due to insufficient precision when determining the center locations and the pixel shift of microlens subimages. In this paper, we present a correction method for MLA errors with subpixel accuracy. Compared with previous algorithms, the proposed method mainly improves two key techniques of light field correction: feature point extraction and subimage registration. The center location and edge detection algorithms based on multi-interpolation are first used to improve the accuracy of subimage feature-point location from the integer pixel level to subpixel level. This can enhance the sensitivity of the subimage feature variation and obtain the accurate coordinates of the feature points, thereby avoiding the effect of coordinate deviations caused by the integer pixel location on the correction solution. Then the subpixel registration method based on backward mapping and grayscale interpolation is used to realign the geometric-corrected subimages to the ideal positions, avoiding vacant pixels and improper subimage movement due to the forward mapping registration at an integer pixel level. To evaluate the proposed method, we derive a complete error model correlating the MLA form errors with orientation errors and obtain multiple sets of raw images under different error conditions using a ray-tracing simulation plenoptic imaging system [30]. The reasonable upper bounds and correction accuracy of the errors are analyzed by measuring the image quality before and after correction. A benchmark comparison with the previous method demonstrates the superior performance of the proposed method for correcting image distortions.

## 2. Correlated MLA Error Model

**Figure 1:**
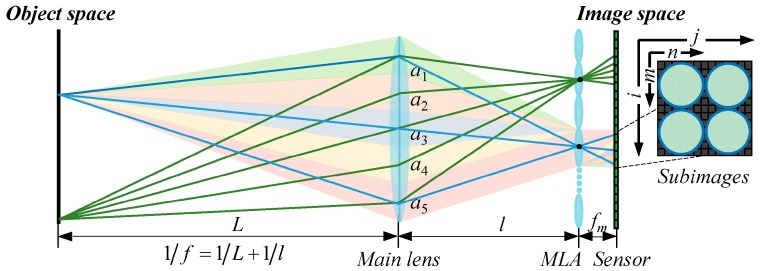
Optical configuration and ray tracing of an unfocused plenoptic imaging system.

In an unfocused plenoptic imaging system, the MLA is positioned at the imaging plane of the main lens and the image sensor is located behind at a distance equal to the focal length of the microlens, as illustrated in Figure 1. The MLA determines the angular sampling distribution of the rays passing through a point at the main lens pupil and projects it onto the sensor pixels assigned to the corresponding microlenses to form subimages. Such a system structure can achieve maximal light-field depth resolution and provide depth constraints to estimate the object depth map without calibration [2,3]. Ideally, each microlens has an identical geometric form and a uniform arrangement, and the MLA is aligned with the image sensor. Thus, a definite correspondence between the object-space points, the microlenses, and the subimage-space points can be established to convert a 2D raw image into 4D light-field data. However, owing to defects arising from manufacturing and assembly adjustment processes, the form and orientation parameters of the MLA may deviate from the design values, resulting in mismatches between the spatial and angular information recorded by the subimages. Many kinds of MLA errors that occur in the imaging system are limited to the use of different machining materials and methods [31,32,33]. According to their characteristics, MLA errors can be classified into two categories: form errors, which include pitch error, radius-of-curvature error, and decenter error; and orientation errors, which comprise distance error, translation error, and tilt error. The actual MLA error can be represented as a combination of these basic error types.

**Figure 2:**
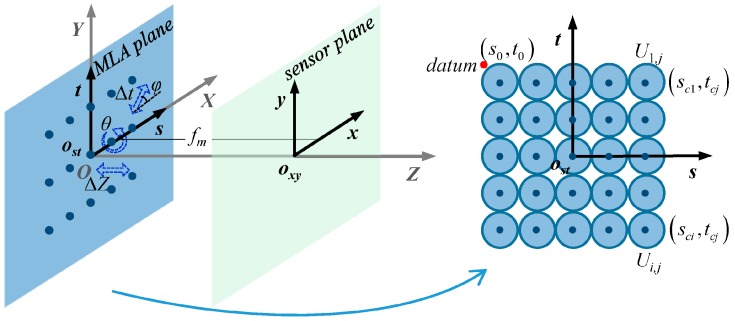
Illustration of the coordinate system in the MLA error model.

MLA errors involve the MLA plane and the image sensor plane of the plenoptic system. To describe the error models, we establish a coordinate system *O*-*XYZ* between the two parallel planes as shown in Figure 2, including an MLA coordinate *o_st_*-*st* with its origin at the MLA center and an image sensor coordinate *o_xy_*-*xy* with its origin at, ${\left(0,0,f_{m}\right)}^{T}$ where $f_{m}$ is the focal length of the microlens. The *Z*-axis corresponds to the optical axis of the system, whereas the *X*-and *Y*-axes are parallel to the sensor pixel grids. The origin *O* is at the intersection between the optical axis and the conjugate image plane of the main lens. In the ideal case, the origin *o_st_* coincides with the origin *O*, that is, the origin *o_st_* is at ${\left(0,0,0\right)}^{T}$ with the *s*-and *t*-axes are parallel to the *X*-and *Y*-axes and the *x*-and *y*-axes, respectively. For a planar MLA arranged in a matrix-form [29], which consists of *N_W_* × *N_H_* microlenses in a square arrangement, the length and center pitch of the microlenses are both *p*, the two sides of the microlenses are spherical surfaces with radius-of-curvature *r*, the microlens height is *h*, and the optical axes of the microlenses are parallel to *Z*-axis. The orientation of an arbitrary microlens *U_i,j_* in *o_st_*-*st* coordinates can be represented by its center point as ${\left(s_{ci},t_{cj}\right)}^{T}={\left(s_{0}+\left(i-1/2\right)p,t_{0}-\left(j-1/2\right)p\right)}^{T}$, where *i* and *j* denote the microlens in the *i*-th row and the *j*-th column of the MLA, $i=1,2,\cdots ,N_{H}$ and $j=1,2,\cdots ,N_{W}$, and ${\left(s_{0},t_{0}\right)}^{T}={\left(-N_{W}p/2,N_{H}p/2\right)}^{T}$ is the orientation-datum coordinates of the MLA plane. Accordingly, the geometric form of each microlens can be described as symmetrical spherical surfaces:

(1) $${\left(s-s_{ci}\right)}^{2}+{\left(t-t_{cj}\right)}^{2}+{\left[Z\mp \left(r-h/2\right)\right]}^{2}=r^{2}$$

where the negative or positive sign for the term (r−h/2) denotes the entrance or exit side of the microlens.

If the MLA is perfectly aligned, that is, the coordinates *o_st_*-*st* are on the plane *O*-*XY*, only the first category errors have to be considered. We define the microlens form errors in a unit or region under the condition that the other parameters are constant and use Δ*p*, Δ*r* and *δ* to represent the pitch deviation, radius-of-curvature deviation, and non-collinear offset of the spherical centers on both sides of the microlens, respectively. The corresponding mathematical models can be deduced in accordance with Equation (1) and the results are listed in Table 1, where *α* and *β* are the angles of the pitch error Δ*p* and the decenter error *δ*, respectively, relative to the horizontal direction.

Then we discuss the MLA orientation error models and formulate the transformation matrix between the misaligned plane *o_sʹtʹ_*-*sʹtʹ* and the ideal plane *o_st_*-*st*. Assume that the image sensor plane *o_xy_*-*xy* shown in Figure 2 is mounted on and along its ideal position and direction, and this plane is used as an orientation reference. As shown in Figure 2, if the MLA plane moves along the *Z* axis, which changes the coupling distance from the main lens and the image sensor, the resulting error is referred to as the coupling distance error Δ*z*. This error can destroy the conjugate imaging conditions of the plenoptic system, thereby reducing its light-field resolution and focusing-depth range. If the MLA plane moves on plane *O*-*XY*, that is, it undergoes a translation perpendicular to the *Z* axis, the resulting error is referred to as the translation error Δ*t*, and *φ* is the angle of the error relative to the horizontal direction. The translation error causes all the microlens subimages to be offset in the direction of the error, resulting in a shift in the recorded spatial information of the target. If the MLA plane is not parallel to the image sensor plane and there is a tilt angle around the *t*-axis, the resulting error is referred to as tilt error *θ*. Because of this tilt angle, the optical axis of each microlens is also tilted, and the coupling distance varies linearly with the distance from each microlens to the horizontal axis of the MLA, which makes the subimages appear as different defocusing and position offsets. The MLA plane *o_sʹtʹ_*-*sʹtʹ* with orientation errors can be obtained via translation and rotation transformation of plane *o_st_*-*st*. The two planes satisfy the relationship:

(2) $$\left[\begin{array}{c} s^{\prime }\\ t^{\prime }\\ z^{\prime }\\ 1 \end{array}\right]=\left[\begin{array}{cc} R_{t} & T\\ 0^{T} & 1 \end{array}\right]\left[\begin{array}{c} s\\ t\\ 0\\ 1 \end{array}\right]$$

where $T={\left(\Delta t\cos\varphi ,\Delta t\sin\varphi ,\Delta z\right)}^{T}$ is the translation matrix comprising error parameters Δ*t* and Δ*z*, and $R_{t}$ is the rotation matrix around the *t* axis. The latter is associated with the error angle *θ*, which can be represented as:

(3) $$R_{t}=\left[\begin{array}{ccc} \cos\theta & 0 & \sin\theta \\ 0 & 1 & 0\\ -\sin\theta & 0 & \cos\theta \end{array}\right]$$

By combining Equations (2) and (3), we can obtain the center coordinates ${\left({s^{\prime }}_{ci},{t^{\prime }}_{cj},z^{\prime },1\right)}^{T}$ of the microlens *U_i,j_* in *O*-*XYZ* coordinates under the specified orientation error conditions as follows:

(4) $$\left[\begin{array}{c} {s^{\prime }}_{ci}\\ {t^{\prime }}_{cj}\\ z^{\prime }\\ 1 \end{array}\right]=\left[\begin{array}{cccc} \cos\theta & 0 & \sin\theta & \Delta t\cos\varphi \\ 0 & 1 & 0 & \Delta t\sin\varphi \\ -\sin\theta & 0 & \cos\theta & \Delta z\\ 0 & 0 & 0 & 1 \end{array}\right]\left[\begin{array}{c} s_{ci}\\ t_{cj}\\ \pm \left(r-h/2\right)\\ 1 \end{array}\right]$$

At this point, the corresponding surface equation can be also obtained by substituting the results of Equation (4) in Equation (1). Consequently, the MLA orientation errors can be combined with the microlens form errors to construct a correlated MLA error model, in which the parameters for each category and type of error are independent.

## 3. Correction Method

### 3.1. Principle

During the light-field recording, the form and arrangement of the microlenses govern the spatial distribution of the subimages, and the orientation of the MLA determines the sampling relationship of the spatio-angular information of light rays. Whenever the MLA incurs in form or orientation error, the subimages get mapped into a distorted image on the sensor plane. The observed distortion is predominantly of geometric distortion caused by translation, rotation and scaling of subimages, and grayscale distortion, such as blurred edges and changing brightness and contrast of images. Thereby, these distortions can be described using geometric and grayscale transformations. For a given feature point $x_{i,j}$ on the 2D image plane and its corresponding ideal point ${x^{\prime }}_{i,j}$, the geometric transformation between them is given by:

(5) $$x_{i,j}={P^{\prime }}_{i,j}{x^{\prime }}_{i,j}$$

where subscripts *i* and *j* represent the subimage in the *i*-th row and the *j*-th column of the distorted image, and $x_{i,j}={\left(x_{i,j},y_{i,j},1\right)}^{T}$ and ${x^{\prime }}_{i,j}={\left({x^{\prime }}_{i,j},{y^{\prime }}_{i,j},1\right)}^{T}$ are the homogeneous coordinates of the feature point in subimage i,j and the corresponding point in the ideal subimage, respectively. ${P^{\prime }}_{i,j}$ is the geometric error matrix of subimage i,j which contains translation, rotation, and scaling transformations and is defined as:

(6) $${P^{\prime }}_{i,j}=\left[\begin{array}{cc} R_{i,j} & t_{i,j}\\ 0^{T} & 1 \end{array}\right]=\left[\begin{array}{ccc} r_{i,j}^{11} & r_{i,j}^{12} & t_{i,j}^{13}\\ r_{i,j}^{21} & r_{i,j}^{22} & t_{i,j}^{23}\\ 0 & 0 & 1 \end{array}\right]$$

where $R_{i,j}$ is the rotation scaling matrix of subimage i,j and $t_{i,j}$ is the translation vector. Because the coordinates of the feature points in the distorted light-field image are known, we can obtain the undistorted pixel point ${x^{\prime }}_{i,j}$ from the pixel point $x_{i,j}$ in the captured distorted subimage via geometric transformation with ${P^{\prime }}_{i,j}$. It is clear that $R_{i,j}$ is a nonsingular matrix, then ${P^{\prime }}_{i,j}$ is also a nonsingular matrix. Hence, there is an inverse matrix $P_{i,j}={\left({P^{\prime }}_{i,j}\right)}^{-1}$ such that:

(7) $${x^{\prime }}_{i,j}={\left({P^{\prime }}_{i,j}\right)}^{-1}x_{i,j}=P_{i,j}x_{i,j}$$

where $P_{i,j}$ is the geometric correction matrix of subimage *i*,*j*. It can be concluded from Equation (6) that $P_{i,j}$ has six degrees of freedom and that, theoretically, can be estimated using three feature point pairs of $x_{i,j}$ and ${x^{\prime }}_{i,j}$ within the subimage. However, to improve solution accuracy and reduce computational complexity and time, we consider five feature points, namely the calibrated center point and four edge points along the center lines of each subimage, and perform geometric correction according to Equation (7).

After that, the grayscale distortion in subimage i,j is corrected. This process serves two considerations: on the one hand, rectifying the brightness and contrast deviations in the subimages caused by the MLA errors and improving the overall grayscale uniformity of the light-field image, and, on the other hand, eliminating the additional grayscale inconsistencies, especially those concerning uneven pixel intensity after the scaling transformation, induced during geometric correction. The purpose of the grayscale correction is to adjust each subimage to the equivalent intensity level of the ideal subimage so as to guarantee the accuracy of the light-field data and the reconstruction results. We define the grayscale correction factor for subimage *i*,*j* after geometric correction as:

(8) $$g_{i,j}=\frac{\mu_{i,j}^{u}}{\mu_{i,j}^{d}}$$

where $\mu_{i,j}^{d}$ and $\mu_{i,j}^{u}$ are the average intensities of the grayscale-distorted subimage *i*,*j* and the its undistorted subimage, respectively. Thus, using the grayscale correction factor $g_{i,j}$, we can transform the intensity value $f_{i,j}\left(x^{\prime },y^{\prime }\right)$ of the pixel point $\left(x^{\prime },y^{\prime }\right)$ in geometric-corrected subimage *i*,*j* by Equation (9) to obtain the corrected intensity value ${f^{\prime }}_{i,j}\left(x^{\prime },y^{\prime }\right)$:

(9) $${f^{\prime }}_{i,j}\left(x^{\prime },y^{\prime }\right)=g_{i,j}f_{i,j}\left(x^{\prime },y^{\prime }\right)$$

From the above analysis, once the spatial geometric and grayscale information of the distorted subimage *i*,*j* and the corresponding ideal subimage are known, it is possible to estimate the desired coordinates of each pixel point using the geometric correction matrix $P_{i,j}$ and its intensity values obtained using the grayscale correction factor $g_{i,j}$ to restore the 4D light field data recorded by the subimage. In our previous method, the solution accuracy of $P_{i,j}$ suffered from the integer-pixel extraction for the subimage feature points where the center and edge points could only reach integer pixel points. Another problem was that the geometric transformation process for subsequent subimage correction operated using integer pixel units, which caused deviations in the geometric and grayscale characteristics of some pixels after correction. Therefore, in this paper, we apply feature point extraction with subpixel accuracy and subpixel registration based on backward mapping to correct light-field images.

### 3.2. Subpixel Feature-Point Extraction

In order to accurately determine the error and correction matrices derived in Section 3.1, we propose a subpixel-level method for the extraction of feature points from raw images. This method can locate the center points and edge feature points of each microlens subimage and divide the corresponding subimage regions, allowing for better-performing local geometric and grayscale corrections on the light-field image, which will be described in the next section. The extraction procedure is summarized in Algorithm 1.

**Algorithm 1** Feature-Point Extraction Procedures 
Capture a white light-field raw image
Bilateral filtering
Segment microlens subimage regions by the threshold method
1: **Procedure** Center-Point Estimation
  Compute the overall intensity of the pixels within each row and column of the subimage regionEquation (10)  Determine coarse center regionsEquation (11)  Subdivided center regions by bilinear interpolationEquation (12)  Estimate the coordinates of the center point at a subpixel level based on a center-of-gravity algorithmEquation (13)2: **Procedure** Edge-Point Estimation
  Detect edge pixels using Sobel operator and polynomial interpolationEquations (14)–(16)  Fit defined parabolic functions ϕ(x) and ϕ(y) to edge pixels and adjacent pixels Equation (17)  Estimate the coordinates of the edge point at a subpixel levelEquation (18)

**Figure 3:**
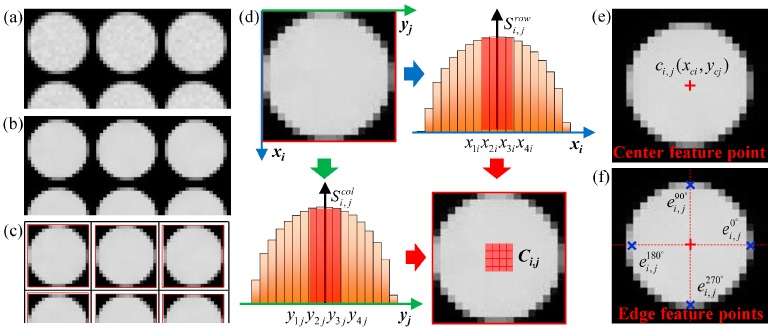
Location and extraction of subpixel feature points: (**a**) White raw image; (**b**) Bilateral-filtered image; (**c**) Microlens subimage region segments; (**d**) Coarse center region location; (**e**) Extracted center feature point; and (**f**) Extracted edge feature points.

First, a white raw image of an evenly illuminated flat white surface is captured, an example of which is shown in Figure 3a. This image contains geometric and grayscale information of all the microlens subimages. To reduce signal noise, the captured image is preprocessed by bilateral filtering. A bilateral filter considers both the spatial proximity and grayscale similarity between two pixel points and can preserve the edge information. The filtered counterpart of the image is presented in Figure 3b. Then, we threshold the image, which is afterwards segmented it into a series of distinct subimage regions. The threshold value depends on the overall intensity of the image pixels. The sum of the intensity values within each row and each column of the filtered image is calculated as follows:

(10a) $$S^{row}\left(x\right)=\sum_{y=1}^{N}f\left(x,y\right),x=1,2,\cdots ,M$$

(10b) $$S^{col}\left(y\right)=\sum_{x=1}^{M}f\left(x,y\right),y=1,2,\cdots ,N$$

where S(⋅) denotes the sum of intensity values, superscripts *row* and *col* denote summing by rows and by columns, respectively, f(x,y) is the intensity value of the pixel point (x,y), and *M* and *N* correspond to resolution of the image, which is *M* (H) × *N* (W). Threshold values of $Th^{row}=15\underset{1\leq x\leq M}{\min}S^{row}\left(x\right)$ and $Th^{col}=15\underset{1\leq y\leq N}{\min}S^{col}\left(y\right)$ are used for the segment boundaries; the *x*-th row with $S^{row}\left(x\right)$ less than $Th^{row}$ is identified as the horizontal boundary, and likewise, the *y*-th column with $S^{col}\left(y\right)$ less than $Th^{col}$ is identified as the vertical boundary. The microlens subimage regions $R:R_{1,1},R_{1,2},\ldots ,R_{i,j},\ldots$ are then determined according to the adjacent boundaries as shown in Figure 3c, where subscripts *i* and *j* represent the subimage in the *i*-th row and the *j*-th column. The subpixel extraction of feature points is based on these segmentation results. Next, we estimate the center feature points of each microlens subimage. In accordance with the vignetting effect, the microlens centers can be identified by the peak intensity points in the subimages. To mitigate the impact of uneven diffuse reflection on center calibration, the pixel intensities over the entire subimage region are gathered and summed along each row and column, that is:

(11a) $$S_{i,j}^{row}(x)=\sum_{y=n_{i,j}^{b}}^{n_{i,j}^{e}}f(x,y),x=m_{i,j}^{b},\cdots ,m_{i,j}^{e}$$

(11b) $$S_{i,j}^{col}(y)=\sum_{x=n_{i,j}^{b}}^{n_{i,j}^{e}}f(x,y),y=n_{i,j}^{b},\cdots ,n_{i,j}^{e}$$

where *m* and *n* denote the row and column numbers of the horizontal and vertical boundaries of subimage region $R_{i,j}$, respectively, and superscripts *b* and *e* represent the beginning and ending boundaries of the subimage region. The rows and columns that correspond to the four highest $S_{i,j}^{row}$ and $S_{i,j}^{col}$ values are sorted in ascending order. The components of the sorted rows and columns are labelled as $x_{1i},x_{2i},x_{3i},x_{4i}$ and $y_{1j},y_{2j},y_{3j},y_{4j}$, respectively, to constitute a 4 × 4 coarse center region $C_{i,j}=\{(x_{1i},y_{1j}),(x_{1i},y_{2j}),\cdots ,(x_{2i},y_{1j}),(x_{2i},y_{2j}),\cdots ,(x_{4i},y_{4j})\}$, as shown in Figure 3d. To achieve subpixel accuracy for the center location, bilinear interpolation with a coefficient of *k* is performed to subdivide the pixels in the coarse center region $C_{i,j}$. Let $\left(x+\tilde{p},y+\tilde{q}\right)$ denote an interpolation point, and its intensity value is determined by the adjacent integer pixel points (x,y), (x+1,y), (x,y+1) and (x+1,y+1):

(12) $$\begin{array}{c} f(x+\tilde{p},y+\tilde{p})=(1-\tilde{p})(1-\tilde{p})f(x,y)+\tilde{p}(1-\tilde{p})f(x,y+1)+\\ \;\tilde{p}\;(1-\tilde{p})f(x+1,y)+\tilde{p}\tilde{p}f(x+1,y+1) \end{array}$$

where $\tilde{p}=p/k$ and $\tilde{q}=q/k$ are the row and column spacing from the integer pixel point (x,y), and $0<\tilde{p}<1$ and $0<\tilde{q}<1$. Here, *p* and *q* denote the interpolation point in the *p*-th row and *q*-th column between adjacent integer pixel points, p=1,2,⋯,k−1 and q=1,2,⋯,k−1. Finally, we estimate the subpixel center point $\left(x_{ci},y_{cj}\right)$ of the subimage based on a center-of-gravity algorithm:

(13) $$\left\{ \begin{array}{l} x_{ci}=\sum_{x=1}^{4k}\sum_{y=1}^{4k}xf(x,y)/\sum_{x=1}^{4k}\sum_{y=1}^{4k}f(x,y)\\ y_{cj}=\sum_{x=1}^{4k}\sum_{y=1}^{4k}yf(x,y)/\sum_{x=1}^{4k}\sum_{y=1}^{4k}f(x,y) \end{array}\right.$$

The estimated result is shown in Figure 3e, where the red cross-point is the center feature point.

The last step of the proposed method is to extract the edge feature points from the subpixel center lines (i.e., the $x_{ci}$-th row and $y_{cj}$-th column where the center point is located). Considering the image features and edge location requirements, we use an edge detection method combined with Sobel templates and polynomial interpolations. The Sobel template is used to detect pixel points in the horizontal and vertical gradient directions of the calibrated center point and initially locate edge points at the integer pixel points $E_{i,j}^{0{}^{\circ}}$ and $E_{i,j}^{180{}^{\circ}}$ as well as $E_{i,j}^{90{}^{\circ}}$ and $E_{i,j}^{270{}^{\circ}}$ along each direction, namely:

(14a) $$E_{i,j}^{0{}^{\circ}}(x_{ci},Y_{1j})=\underset{y_{cj}\leq y\leq n_{i,j}^{e}}{\arg\max}\left\{g_{0{}^{\circ}}(x_{ci},y)\right\}$$

(14b) $$E_{i,j}^{180{}^{\circ}}(x_{ci},Y_{2j})=\underset{n_{i,j}^{b}\leq y<y_{cj}}{\arg\max}\left\{g_{180{}^{\circ}}(x_{ci},y)\right\}$$

(14c) $$E_{i,j}^{90{}^{\circ}}(X_{1i},y_{cj})=\underset{m_{i,j}^{b}\leq x<x_{cj}}{\arg\max}\left\{g_{90{}^{\circ}}(x,y_{cj})\right\}$$

(14d) $$E_{i,j}^{270{}^{\circ}}(X_{2i},y_{cj})=\underset{x_{cj}\leq x\leq m_{i,j}^{b}}{\arg\max}\left\{g_{270{}^{\circ}}(x,y_{cj})\right\}$$

where $g_{0{}^{\circ}}(x_{ci},y)$, $g_{180{}^{\circ}}(x_{ci},y)$, $g_{90{}^{\circ}}(x,y_{cj})$, and $g_{270{}^{\circ}}(x,y_{cj})$ are the convolution results for the Sobel templates in gradient directions of 0°, 180°, 90°, and 270°, respectively. The convolutions can be calculated as:

(15a) $$\begin{array}{cc} g_{0{}^{\circ}}(x_{ci},y)= & f(x_{ci}-1/k,y+1)+2f(x_{ci},y+1)+f(x_{ci}+1/k,y+1)\\ & -f(x_{ci}-1/k,y-1)-2f(x_{ci},y-1)-f(x_{ci}+1/k,y+1) \end{array}$$

(15b) $$\begin{array}{cc} g_{180{}^{\circ}}(x_{ci},y)= & f(x_{ci}-1/k,y-1)+2f(x_{ci},y-1)+f(x_{ci}+1/k,y+1)\\ & -f(x_{ci}-1/k,y+1)-2f(x_{ci},y+1)-f(x_{ci}+1/k,y+1) \end{array}$$

(15c) $$\begin{array}{cc} g_{90{}^{\circ}}(x,y_{cj})= & f(x+1,y_{cj}-1/k)+2f(x+1,y_{cj})+f(x+1,y_{cj}+1/k)\\ & -f(x-1,y_{cj}-1/k)-2f(x-1,y_{cj})-f(x-1,y_{cj}+1/k) \end{array}$$

(15d) $$\begin{array}{cc} g_{270{}^{\circ}}(x,y_{cj})= & f(x-1,y_{cj}-1/k)+2f(x-1,y_{cj})+f(x-1,y_{cj}+1/k)\\ & -f(x+1,y_{cj}-1/k)-2f(x+1,y_{cj})-f(x+1,y_{cj}+1/k) \end{array}$$

For the purpose of the high-accuracy edge locations, the pixel points at 1/*k* from the center lines are used as the adjacent pixel points in the convolution calculations, and the intensity values are obtained via polynomial interpolation. Let $\left(\tilde{x},y\right)$ be a non-integer pixel point and $\left(x_{0},y\right)$, $\left(x_{1},y\right)$, $\left(x_{2},y\right)$ and $\left(x_{3},y\right)$ be adjacent integer pixel points that satisfy $x_{0}<x_{1}<\tilde{x}<x_{2}<x_{3}$. We define an interpolation function $\varphi (\tilde{x})$ as:

(16) $$\varphi (\tilde{x})=\sum_{\alpha =0}^{3}\prod_{\alpha =0,\beta \neq \alpha }^{3}\frac{\tilde{x}-x_{\alpha }}{x_{\alpha }-x_{\beta }}f(x_{\alpha },y)$$

where $\varphi (\tilde{x})$ is the intensity value $f(\tilde{x},y)$ of the pixel point $\left(\tilde{x},y\right)$. Similarly, the intensity value $f(x,\tilde{y})$ can be obtained using an interpolation function $\varphi (\tilde{y})$. For the initial edge points $E_{i,j}^{0{}^{\circ}}$ and $E_{i,j}^{180{}^{\circ}}$ as well as $E_{i,j}^{90{}^{\circ}}$ and $E_{i,j}^{270{}^{\circ}}$, three adjacent pixel points are taken as interpolation nodes along the gradient direction. Substituting them into interpolation function ϕ(y) in Equation (17a) or interpolation function ϕ(x) in Equation (17b), the subpixel edge points along the horizontal and vertical directions can be determined by considering dϕ(y)/dy=0 or dϕ(x)/dx=0:

(17a) $$\phi (y)=\sum_{\alpha =0}^{2}\prod_{\alpha =0,\beta \neq \alpha }^{2}\frac{y-y_{\alpha }}{y_{\alpha }-y_{\beta }}g(x,y_{\alpha })$$

(17b) $$\phi (x)=\sum_{\alpha =0}^{2}\prod_{\alpha =0,\beta \neq \alpha }^{2}\frac{x-x_{\alpha }}{x_{\alpha }-x_{\beta }}g(x_{\alpha },y)$$

Here, $g(x,y_{\alpha })$ and $g(x_{\alpha },y)$ are the gradient values of the interpolation nodes along the corresponding gradient directions, which can be calculated using Equation (15). Considering $E_{i,j}^{0{}^{\circ}}(x_{ci},Y_{wj})$ for example, the interpolation nodes are $\left(x_{ci},Y_{wj}-1\right)$, $\left(x_{ci},Y_{wj}\right)$, and $\left(x_{ci},Y_{wj}+1\right)$ for the interpolation function ϕ(y); thus, the subpixel coordinate $y_{1j}$ can be expressed as

(18) $$y_{1j}=Y_{1j}+\frac{g(x_{ci},Y_{1j}-1)-g(x_{ci},Y_{1j}+1)}{2\left[g(x_{ci},Y_{1j}-1)-2g(x_{ci},Y_{1j})+g(x_{ci},Y_{1j}+1)\right]}$$

By the above operations on the initial edge points, the final subpixel feature points $e_{i,j}^{0{}^{\circ}}(x_{ci},y_{1j})$, $e_{i,j}^{180{}^{\circ}}(x_{ci},y_{2j})$, $e_{i,j}^{90{}^{\circ}}(x_{1i},y_{cj})$, and $e_{i,j}^{270{}^{\circ}}(x_{2i},y_{cj})$ within the subimage region $R_{i,j}$ are obtained, as shown in Figure 3f, where the blue cross-points are the edge feature points.

### 3.3. Geometric and Grayscale Correction

With the feature-point pairs of subimage region $R_{i,j}$ and its ideal subimage, which is estimated as explained in Section 3.2, the geometric correction matrix $P_{i,j}$ can be accordingly worked out, so that the distorted subimages can be then corrected and re-registered via coordinate transformation and mapping. We use a backward model for inverse mapping from a corrected image to a distorted image, which avoids the occurrence of overlapping or vacant pixels and the excessive computational cost associated with the forward model. For a corrected subimage ${R^{\prime }}_{i,j}$, the pixel point $\left({x^{\prime }}_{i,j},{y^{\prime }}_{i,j}\right)$ is mapped back to $\left(x_{i,j},y_{i,j}\right)$ in the distorted subimage $R_{i,j}$ in accordance with Equation (7), and the intensity value of this mapped point, $f({x^{\prime }}_{i,j},{y^{\prime }}_{i,j})$, can be estimated via bilinear interpolation:

(19) $$\begin{array}{cc} f({x^{\prime }}_{i,j},{y^{\prime }}_{i,j})= & (1-u)(1-v)f(X_{i,j},Y_{i,j})+v(1-u)f(X_{i,j},Y_{i,j}+1)+\\ & u(1-v)f(X_{i,j}+1,Y_{i,j})+uvf(X_{i,j}+1,Y_{i,j}+1) \end{array}$$

where $u=x_{i,j}-X_{i,j}$ and $v=y_{i,j}-Y_{i,j}$ are the decimal fractions of the mapped point $\left(x_{i,j},y_{i,j}\right)$. After subpixel registration, the average intensities of subimage ${R^{\prime }}_{i,j}$ and its ideal subimage are calculated, and the grayscale correction factor $g_{i,j}$ is obtained by Equation (8).

Thus, using the distorted raw white image and its ideal raw white image, the geometric correction matrices and the grayscale correction factors of all the microlens subimages can be estimated to constitute a geometric correction matrix **P** and a gray correction matrix **G** for the light-field raw image. After that, we can apply them to other light-field images captured by the same plenoptic camera. For a raw image **x** to be corrected, the geometric distortion is first removed using **P**, which yields image **x**′ after subimage registration. Afterwards, grayscale transformation is carried out using **G** to realize intensity adjustments, thereby obtaining the desired image **x**″:

(20) $$x^{\prime \prime }=Gx^{\prime }=G\left(Px\right)$$

## 4. Results and Discussion

In this section, we verify the proposed models and correction method by comparing with previous algorithms and present various experimental results on simulated datasets to assess the performance of the proposed method. The experimental platform used was based on a physics-simulation imaging system for the unfocused plenoptic camera introduced in Section 2. Information on the main parameters and properties of this system can be found in a previous work [16]. The simulated light-field raw image had a resolution of 2040 × 2040 pixels with 102 × 102 microlens subimages aligned in a square grid. Each subimage covered a region of 20 × 20 pixels. The ray simulation for the imaging process used the Monte Carlo method and was executed with 10 parallel threads on an Intel(R) Xeon(R) E5-2670v2 server. The total number of computational rays was 3 × 10^10^.

### 4.1. Improved Method Validation

In order to test the effectiveness and feasibility of the improved method proposed in this paper, we employed it to correct the microlens form errors for a direct comparison with the previous method [29]. Distorted white raw images were captured using the simulation imaging system, for which a form error model was set up. The imaging object was a flat and evenly illuminated white plate with a size of 0.25 m × 0.25 m and placed 2.5 m away the main lens. For the image correction process, we set the subpixel interpolation coefficient *k* = 10 in Section 3.2 to achieve high accuracy and efficiency when determining the subimage feature points. We extracted the subimage feature points and estimated the correction matrices for the distorted white raw images using the proposed method. We then projected the matrices onto these white images to verify the correction accuracy of our approach. To benchmark image quality before and after correction, deviations relative to the ideal image were characterized with peak signal-to-noise ratio (PSNR). Table 2 shows the correction results obtained using the previous [29] and presently proposed method for different form error types and values.

We find that the proposed method is more stable and exhibits better compensation effect on each distorted image than the approach reported in [27]. The PSNR values of the corrected raw images with pitch and decenter errors exceed 29.6 dB and 28.9 dB, respectively. For a relative radius-of-curvature error ranging from −5% to 10%, the PSNR values of the images after grayscale correction are over 30.2 dB with negligible distortion. This indicates a significant improvement in correction performance compared with the previous results. The geometric correction accuracy is raised by a maximum of 17.5% and by 8.4% on average, and grayscale correction accuracy is raised by a maximum of 16.0% and by 7.1% on average. Additionally, the overall accuracy is observed to remain nearly constant as the error value increases. The main reason for this enhancements in accuracy stem from deduction of a more accurate geometric correction matrix based on subpixel feature points as well as subpixel registration based on multiple interpolations, thus eliminating mapping deviations and the inhomogeneous intensities of pixel points in the correction transformations.

**Figure 4:**
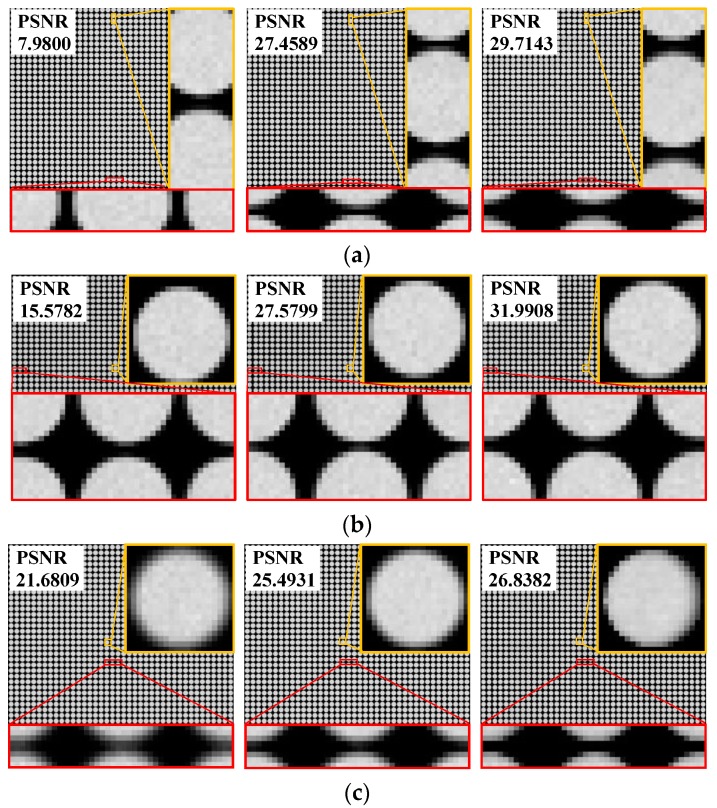
Comparison between partially enlarged white raw images (**a**) with a pitch error of Δ*p* = 1.0 μm at *α* = 90°, (**b**) with a decenter error of *δ* = 10 at *β* = 90°, and (**c**) with a radius of curvature error of *ε_r_* = −10% before and after correction. Left: distorted image; Center: corrected image using the previous method [27]; Right: corrected image using the proposed method. The PSNR values of each case are displayed in the upper left corner of the image.

The distorted raw white images under each of the error conditions and the corrected images obtained using both abovementioned methods are shown in Figure 4. It can be observed from the partially enlarged views of Figure 4a,b that, in regards to the subimage shifts caused by the pitch and decenter errors, the proposed method can realign each subimage and only slight position deviations retain. After correction, the horizontal and vertical pitches between adjacent subimages are consistent with the ground truths (see the red frames), and the microlens subimages are symmetrical in shape with no deformation (see the yellow frames). As shown in the images with radius-of-curvature error in Figure 4c, the blurring and aliasing of the subimages is clearly alleviated by our method, which produces smoother edges, more homogeneous brightness, and enhanced contrast compared with the original distorted images and the images corrected with the previous method. These details within gray-image further indicate that the proposed correction method is superior to the previous algorithm.

### 4.2. Orientation Error Correction Effect

When using commercial plenoptic cameras or self-assembly prototype cameras for measurements, small orientation errors may exist in the MLA owing to pre-adjustment deviations in the imaging system and to vibrations and high temperatures in the surrounding environment. Considering these actual errors and the degradation level of erroneous images, we set the upper bounds of the coupling distance error, translation error, and tilt error as 15 μm, 15 μm, and 1.0°, respectively, in our simulation experiments, so as to analyze the correction accuracy of the proposed method for this error category. For each error, we selected simulated raw white images with different error directions and performed independent correction trials. The PSNR values of each raw image and its microlens subimages were measured before and after correction to quantify the overall and local differences between the raw image and the ideal image.

**Figure 5:**
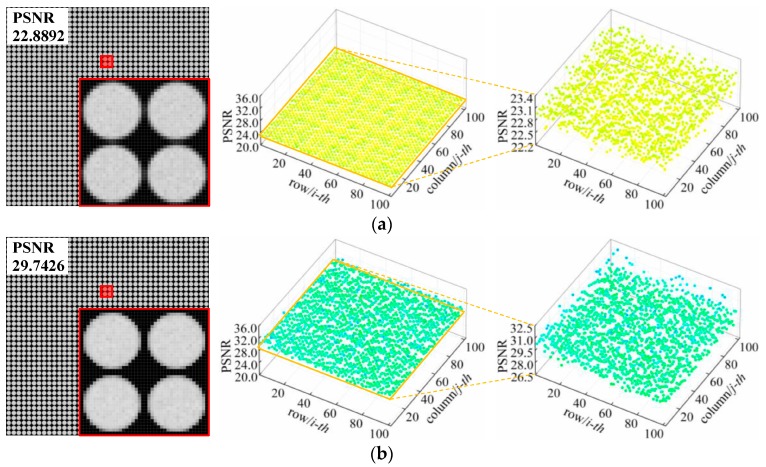

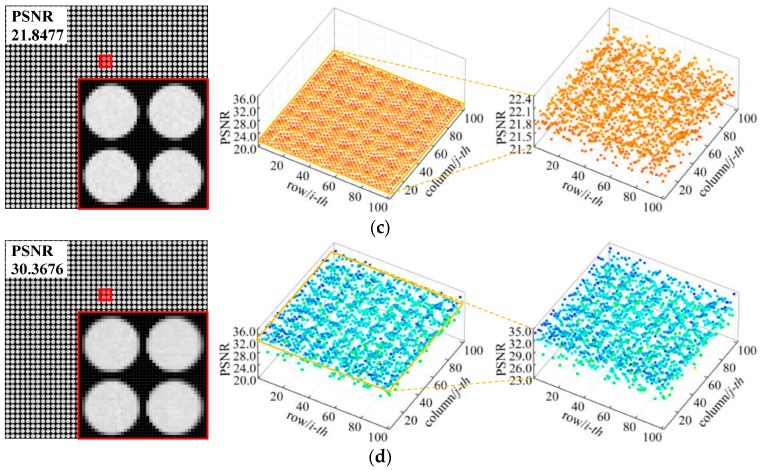
Correction results and deviation distributions for images with coupling distance error: (**a**) Distorted white raw image with an error of Δ*z* = −15 μm and (**b**) its corresponding corrected image; (**c**) Distorted white raw image with an error of Δ*z* = 15 μm and (**d**) its corresponding corrected image. The PSNR values of each case are displayed in the upper left corner of the image.

Firstly, we examined the correction performance with respect to the coupling distance errors in different directions (i.e., with positive and negative values). As shown in the partially enlarged views of Figure 5a,c, this error affects the brightness, size, and edge definition of the subimages. When the coupling distance error is negative, the MLA plane advances such that the light rays that are not fully focused by the main lens diverge through the microlenses, which in turns causes entrance loss and large divergence in the exit rays. As a result, the subimages exhibit low brightness and outward diffusing edges (see the red frame in Figure 5a). In contrast, positive error causes excessive focus and insufficient divergence of light rays and correspondingly, the brightness of the subimages tends to be high along with shrinking edges (see the red frame in Figure 5c). Besides, reference to the overall and local PSNR values of the distorted image reveals that, positive error degrades the quality of light-field images more seriously than negative error for the same error value.

After correcting the light field using the proposed method, the resulting images and correction deviation distributions are shown in Figure 5b,d. As can be observed, the brightness bias and edge scaling in the subimages get refined, and the overall image quality is improved significantly. The PSNR values of the raw images with negative and positive errors increase from 22.89 dB and 21.85 dB to 29.74 dB and 30.37 dB, respectively, whereas the local correction deviation ranges of each subimage are 26.57–32.37 dB and 23.04–35.11 dB. Although there are quality fluctuations in the corrected subimages and those for positive errors are larger, the subimages with PSNR values over 27.50 dB account for 99.93% and 97.25% of the raw images with either negative or positive error, indicating that most subimages are of high quality and almost exactly identical to the corresponding ideal subimages.

**Figure 6:**
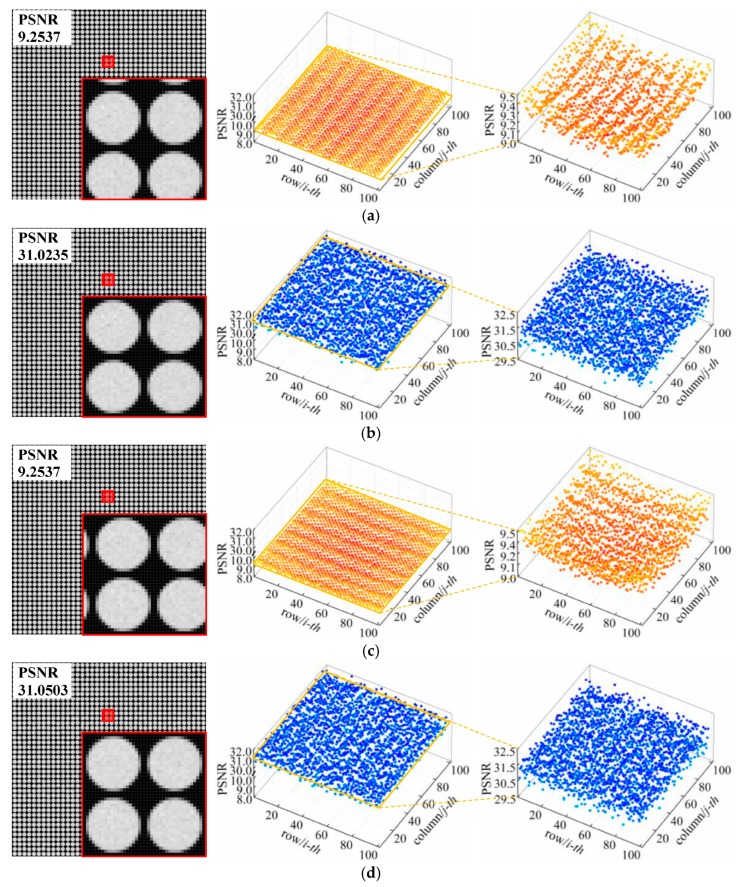
Correction results and deviation distributions for images with translation error: (**a**) Distorted white raw image with an error of Δ*t* = 15 μm at *φ* = 90° and (**b**) its corresponding corrected image; (**c**) Distorted white raw image with an error of Δ*t* = 15 μm at *φ* = 0° and (**d**) its corresponding corrected image. The PSNR values of each case are displayed in the upper left corner of the image.

Secondly, we employed images with vertical and horizontal translation errors to the proposed correction method for translation errors. The feature details of the distorted and corrected images and the numerical comparisons with the ideal subimages are shown in Figure 6. In Figure 6a,c, it can be seen that the subimages of two distorted images are uniform and clear but have a position offset of three pixels along error directions, which led to severe degradation. Consequently, the overall PSNR values of these images are rather low. The translation errors also cause subimage quality to change periodically. The amplitude and period of the local PSNR values for both error direction are roughly the same, and the direction remains in agreement with the error direction.

Upon completion of the correction process, the misalignments of the subimages are eliminated while retaining high-frequency edge information. Moreover, image quality is greatly enhanced, as shown in Figure 6b,d. The PSNR values of the images with vertical and horizontal errors are to 31.02 dB and 31.05 dB, respectively, and those of their corresponding subimages are between 29.73 dB and 32.37 dB with no obvious periodic variations. This demonstrates that the raw images have high quality and that the quality differences between subimages are also small. The presented comparison of images and numerical results confirms the effectiveness of the proposed method as regards translation error correction.

**Figure 7:**
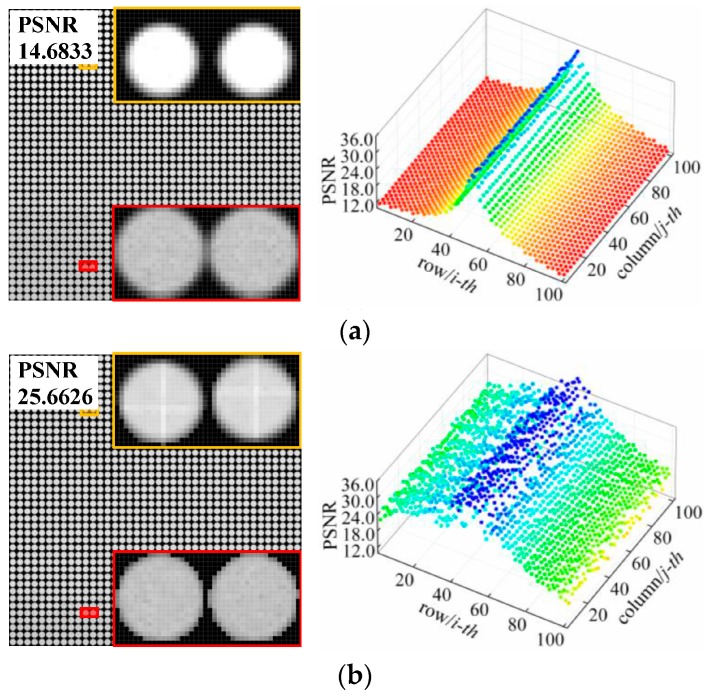
Correction results and deviation distributions for images with tilt error: (**a**) Distorted white raw image with an error of θ = 1.0° and (**b**) its corresponding corrected image. The PSNR values of each case are displayed in the upper left corner of the image.

Thirdly, we used the proposed method to correct the distorted light-field affected by tilt errors. As shown in Figure 7a, the integral distribution of the raw image distortion is symmetric along the horizontal central axis. The subimages in the center row exhibit the smallest distortion and attain the highest PSNR results. The quality of the subimages on either sides decreases dramatically with the increase in row distance from the central axis. It can be seen from the partially enlarged details of the different regions that the subimages are both defocused and shifted downward according to the tilt angle of the MLA plane and the microlens’ optical axis. The difference between the two regions is that the backward offset of the upper microlenses cause the brightness of the subimages to exceed the standard value and a reduction in the number of covering pixels (see the yellow frame). On the other hand, the forward offset of the lower microlenses lowers the brightness of the subimages and induces crosstalk with adjacent subimages (see the red frame).

As for the corrected light-field image, which is shown in Figure 7b, the overall PSNR value of the raw image increase from 14.68 dB to 25.66 dB. The correction deviation range of the subimages in the central portion of the image (rows 21–80) is 24.70–35.96 dB, whereas those in the top (rows 1–20) and bottom (rows 81–102) portions are 23.08–28.69 dB and 15.65–24.67 dB, respectively. Most correction results are close to the ideal case for all subimages; only the PSNR values of the subimages at the edges are low, especially those at the bottom edge, thereby reducing the overall correction accuracy. By referring to the corrected images, we find that the brightness and size of the subimages are recovered to some extent, but at the edges, zigzag-shaped artifacts and discontinuous pixel intensities arise from the scaling transformation. This is the main reason why the correction performance of the proposed method for tilt errors is not as good as that for the other two types of orientation error.

Based on these results and in order to further evaluate the accuracy and stability of the proposed method for correcting orientation errors, we varied the coupling distance and translation errors along two directions from 5 μm to 15 μm at a step of 5 μm and the tilt error from 0.2° to 1.0° at a step of 0.2°. The PSNR results of the distorted raw images under each of the error conditions and the resulting images after geometric and grayscale corrections are listed in Table 3. For the coupling distance error, the PSNR values of the corrected images decrease as the absolute value of the error increases, and correction accuracy reaches over 29.74 dB within the upper error bound. The horizontal and vertical translation errors are observed to degrade the image quality to a similarly low level, and the PSNR results after correction are in the range of 31.01–31.31 dB, which is equivalent to the ideal image. The tilt error correction provides a relatively larger PSNR range, and the PSNR values go from 32.75 to 25.66 dB corresponding to an increase in the error angle. The maximum and average PSNR values have increased by 74.77% and 58.54%, respectively, compared with the uncorrected images. It is noticed that the correction results on the small errors of each orientation error still performs well. Even if the distorted images have high PSNR values with minor geometric and grayscale distortions among the subimages, the quality of corrected images are improved obviously. This indicates that the proposed method is sensitive to the variation of the geometric feature of erroneous sub-images and can locate and extract the coordinates of feature points accurately, thus improving correction performance. Meanwhile, when the error value changes, the PSNR values of the corresponding corrected images are stable at an acceptable level. The correction results shown in Table 3 demonstrate that the proposed method is applicable for realizing correction of orientation errors of different types, directions, and values within a reasonable upper error bound.

### 4.3. Evaluation of Light-Field Correction Performance for Real Scene

**Figure 8:**
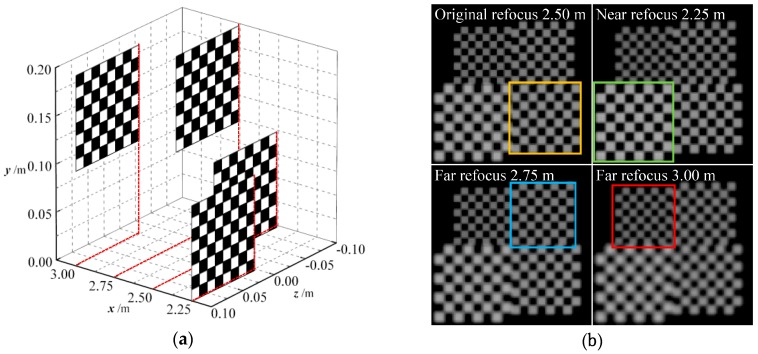
Imaging and correction target for the real scene light field. (**a**) Staggered checkerboards placed at x = 2.25, 2.50, 2.75 and 3.00 m; (**b**) Ideal refocused images at the respective depths.

High-precision correction of light-field raw images is essential in volumetric reconstruction by digital refocusing. In order to verify the accuracy of the light field reconstruction resulting from the proposed correction method, we performed imaging simulations on a real scene as shown in Figure 8a, which consisted of a set of checkerboards placed at 2.5 m from the main lens with a depth interval of 0.25 m. Each checkerboard measured 0.1 m × 0.1 m in size and the unit square side of the checkerboard was 12.5 mm. Figure 8b presents the refocused images of the respective checkerboards that are computed from the ideal light field.

**Figure 9:**
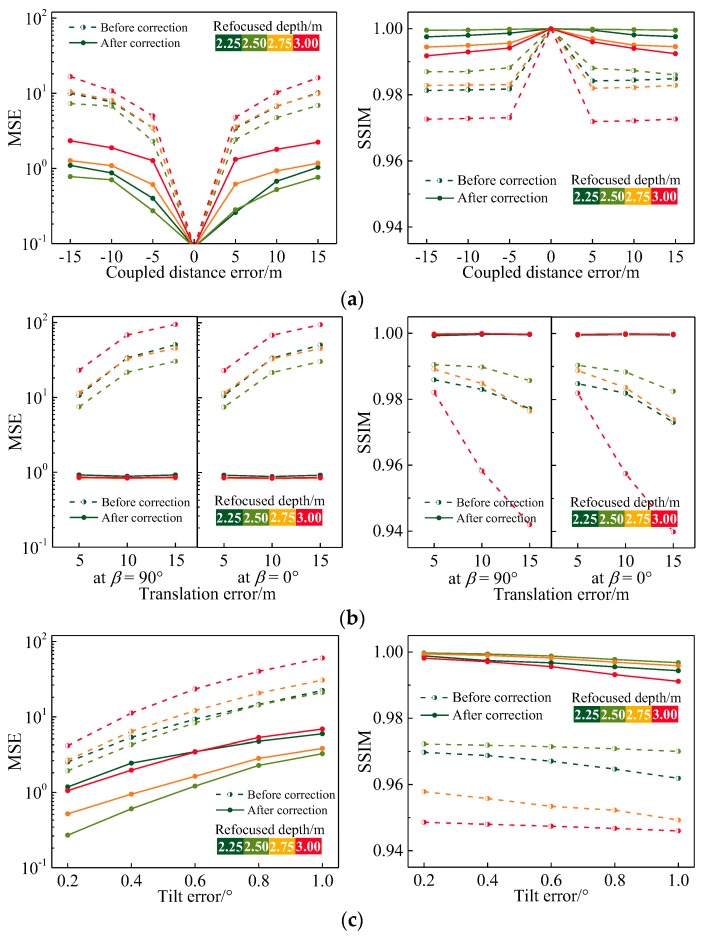
Correction results for light-field refocusing under different orientation error conditions: (**a**) Coupled distance error; (**b**) Translation error; (**c**) Tilt error.

We captured a dataset of distorted real scene images from this setup according to the orientation errors in Table 3 and then performed consecutive corrections on these images using the geometric and grayscale correction matrices of the corresponding errors determined in Section 4.2. For the uncorrected and corrected raw images, we separately generated refocused images at different depths and introduced the mean square error (MSE) and the structural similarity index metric (SSIM) [34] as objective quality measures to characterize the grayscale and structural similarity distortions of the generated images. The MSE based on pixel grayscale is a negative index with a dynamic range of [0,2552] (for 8-bit grayscale images): smaller MSE values indicate smaller average grayscale deviations from the standard image, and MSE = 0 if there is no pixel grayscale error. The SSIM based on image structure is a positive index with a dynamic range of [−1,1]: the higher the SSIM value is, the better the structure similarity of the image; when SSIM = 1, the image has no structural distortion. Figure 9a–c illustrate the quality evaluation results of the light-field refocusing before and after the corresponding correction for the coupling distance errors, translation errors, and tilt errors.

As for the coupling distance error, as shown in Figure 9a, the resultant curves of the uncorrected refocused images (dotted curves) indicate that the refocusing degradations caused by errors in both directions are nearly the same. The MSE values of the refocused images increase as the absolute value of the error increases, and the SSIM values remain constant once the depth of refocus is fixed. As evidenced by the corrected image curves (solid curves), the grayscale and structural similarity of each refocused image shows obvious improvements. The MSE and SSIM values are in the range of 0.2588–2.3331 and 0.9917–0.9998, respectively. As for the translation errors, the results for which are shown in Figure 9b, the objective indexes of the refocused images without correction deteriorate sharply as the error value and the depth of refocus increase. In particular, when the depth of refocus is 3.00 m, the SSIM values of the vertical and horizontal error images reduce from 0.9859 and 0.9848 at Δ*t* = 5 μm to 0.9419 and 0.9398 at Δ*t* = 15 μm, respectively. After the correction process, all the quality evaluation results are within an ideal level and do not vary with the error value or the depth of refocus. The maximum MSE is 0.9859, and the average is 0.9613. The minimum SSIM is 0.9996, and the average is 0.9997. As for the tilt errors, it can be observed from Figure 9c that the MSE range of the refocused images reduces from 1.9375–40.1375 to 0.2714–6.8736 compared with the uncorrected refocused image, whereas the corresponding the SSIM range increases from 0.9459–0.9722 to 0.9911–0.9997. As described above, the proposed correction method can effectively rectify the real scene light field within the specified upper bounds of MLA orientation errors. The light-field refocusing accuracies of coupling distance error, translation error, and tilt error correction are 2.3331, 0.9859, and 6.8736 in terms of MSE, respectively, and 0.9917, 0.9996, and 0.9911 in terms of the SSIM.

**Figure 10:**
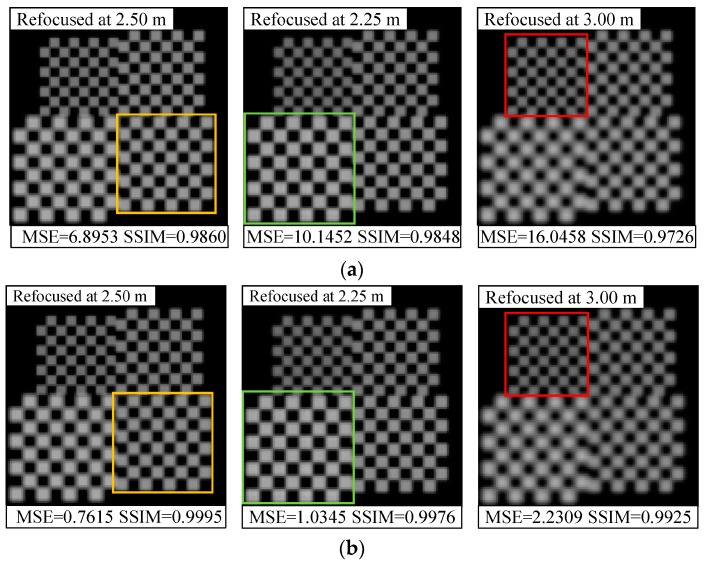
Comparison of refocused images at varying depths for coupled distance error correction: (**a**) With an error of Δ*z* = 15 μm; (**b**) After distortion correction. The MSE and SSIM results for each image are displayed at the bottom.

**Figure 11:**
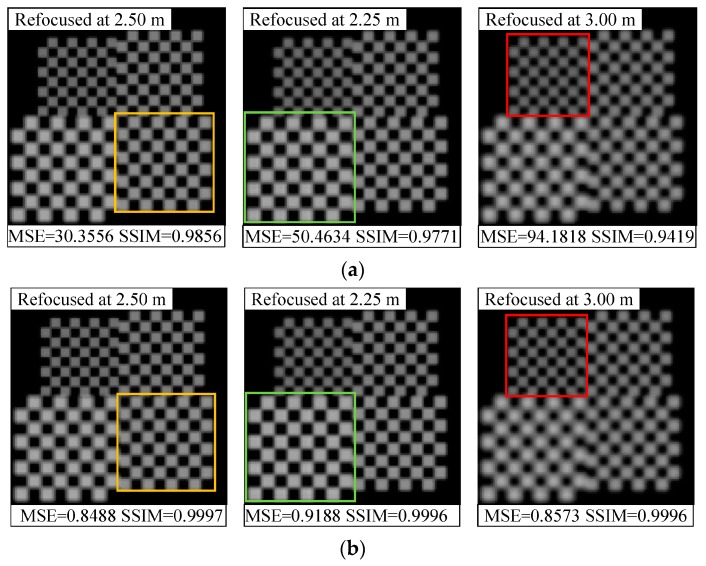
Comparison of refocused images at varying depths for translation error correction: (**a**) With an error of Δ*t* = 15 μm at *φ* = 90 °; (**b**) After distortion correction. The MSE and SSIM results for each image are displayed at the bottom.

**Figure 12:**
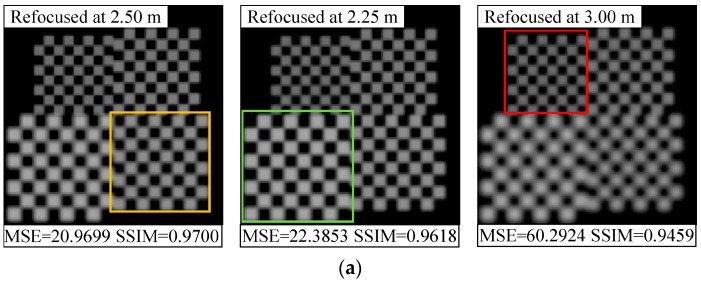

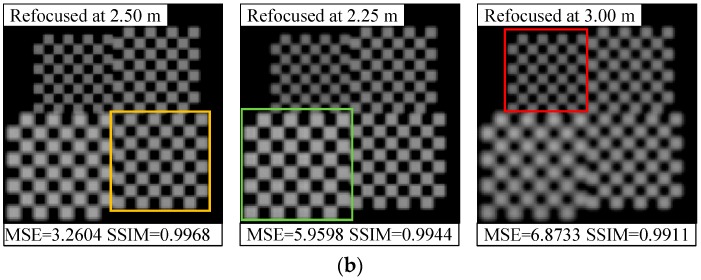
Comparison of refocused images at varying depths for tilt error correction: (**a**) With an error of *θ* = 1.0°; (**b**) After distortion correction. The MSE and SSIM results for each image are displayed at the bottom.

In addition, we compared the refocused images with different depths of the scene before and after orientation error correction, as shown in Figure 10, Figure 11 and Figure 12. We find that the refocused images computed using the uncorrected light fields exhibit similar distortion characteristics to the raw images shown in Section 4.2.

As shown in Figure 10a, the brightness of the refocused image with positive coupling distance error is supersaturated, and the intensity distortion is more evident, along with artifacts at the pattern edges that occurred as the depth of refocus varies. For the vertical translation error shown in Figure 11a, the refocused objects are subject to offset and aliasing distortion along the vertical direction. This extent is the smallest at original focus position (depth of focus of 2.5 m), and downward and upward offsets are observed when refocused closer and refocused further; at the same time, there occurs an increase in the aliasing distortion. As shown in Figure 12a, the pattern is shifted down in the top portion of refocused image, whereas blurring and aliasing appear in the bottom portion. Moreover, the entire image has twisting deformations. This may be attributed to the fact that, the resampled pixels are shifted and superimposed based on the depth of refocus ratio for light-field reconstruction during refocusing. Thus, the distorted subimages of the raw image affect the corresponding refocused image, and the extent of distortion increases as the depth of refocus ratio grows.

On the contrary, with raw light-field correction, and as shown in Figure 10b, Figure 11b, and Figure 12b, the refocused images have less misalignments, distortions, or deformations, and the pattern exhibits sharper edges and more details, which allows for the detection of accurate spatial and intensity information of the object at different depths. These comparison results prove that the proposed method can enhance refocusing effects to obtain refocused images with high quality.

## 5. Conclusions

In this paper, we have presented a geometric and grayscale correction method for MLA errors in plenoptic imaging sensors by directly utilizing raw white images. We formulated a correlated form–orientation error model and derived the correction matrices for the distorted light fields. The proposed correction technique employs subpixel-level feature-point extraction and subimage registration to achieve high correction accuracy and adaptability of the algorithm to MLA errors of different categories and types. Based on the correlated error model, imaging simulations were carried out under given error conditions, and the performance of the proposed correction method was extensively analyzed. A benchmark comparison with the previous method in terms of form error correction indicated that the proposed method achieves significant improvements in accuracy and stability. Geometric and grayscale correction accuracies are increased by 8.4% and 7.1% on average. Performance improvements rely on the more accurate solution for the correction matrices and subimage position realignments, which is obtained by the proposed feature-point extraction algorithms and registration method at a subpixel level. The correction results for orientation error show that, within reasonable upper error bounds, the proposed method is effective and robust for raw images distorted by errors of various types, directions, and values. The PSNR values of the corrected images with coupling distance error, translation error, and tilt error are as high as 29.74 dB, 31.01 dB, and 25.66 dB, respectively. Moreover, we applied the proposed correction method in light-field digital refocusing. Quantitative and qualitative comparisons of the refocused images before and after correction further verify the validity and accuracy of our method and models. The grayscale and structural similarity deviations between the refocused images and the ideal image are both maintained at a low level, which allows for extracting accurate spatial and grayscale data from space objects for subsequent reconstruction and measurement applications.

## Figures and Tables

**Table 1:** Mathematical Model of MLA form error.

Errors	Center Coordinates	Surface Description Equations
Pitch error Δ*p*	$\left(\begin{array}{c} s_{ci}+\Delta p\cos\alpha \\ t_{cj}+\Delta p\sin\alpha \end{array}\right)$	$\begin{array}{l} {\left[s-\left(\left(i-\left(N_{W}+1\right)/2\right)p+\Delta p\cos\alpha \right)\right]}^{2}+{\left[t-\left(\left(\left(N_{W}+1\right)/2-j\right)p+\Delta p\sin\alpha \right)\right]}^{2}\\ +{\left[z\mp \left(r-h/2\right)\right]}^{2}=r^{2} \end{array}$
Radius-of-curvature error Δ*r*	$\left(\begin{array}{c} s_{ci}\\ t_{cj} \end{array}\right)$	$\begin{array}{l} {\left[s-\left(i-\left(N_{W}+1\right)/2\right)p\right]}^{2}+{\left[t-\left(\left(N_{W}+1\right)/2-j\right)p\right]}^{2}\\ +{\left[z\mp \left(r+\Delta r-h/2\right)\right]}^{2}={\left(r+\Delta r\right)}^{2} \end{array}$
Decenter error *δ*	$\left(\begin{array}{c} s_{ci}\\ t_{cj} \end{array}\right)$	$\begin{array}{l} {\left[s-\left(\left(i-\left(N_{W}+1\right)/2\right)p\right)\right]}^{2}+{\left[t-\left(\left(\left(N_{W}+1\right)/2-j\right)p\right)\right]}^{2}\\ +{\left[z-\left(r-h/2\right)\right]}^{2}=r^{2} \end{array}$
	$\left(\begin{array}{c} s_{ci}+\delta \cos\beta \\ t_{cj}+\delta \sin\beta \end{array}\right)$	$\begin{array}{l} {\left[s-\left(\left(i-\left(N_{W}+1\right)/2\right)p+\delta \cos\beta \right)\right]}^{2}+{\left[t-\left(\left(\left(N_{W}+1\right)/2-j\right)p+\delta \sin\beta \right)\right]}^{2}\\ +{\left[z-\left(r-h/2\right)\right]}^{2}=r^{2} \end{array}$

**Table 2:** Quantitative comparison of different correction methods for MLA form errors.

Errors		Distorted Image	Previous [22]		Proposed	
			Geometric Correction	Grayscale Correction	Geometric Correction	Grayscale Correction
Pitch error Δ*p*/μm	0.2	15.2614	27.3087	27.3401	29.7926	29.7975
	0.4	11.3084	26.5720	26.6383	29.6418	29.6524
	0.6	9.5670	27.7079	27.8181	29.6799	29.6992
	0.8	8.5864	28.1185	28.2683	29.6587	29.6842
	1.0	7.9800	27.2738	27.4589	29.6771	29.7143
Radius-of-curvature error *ε_r_*/%	−10	21.6809	22.5709	25.4931	26.5234	26.8382
	−5	27.3614	28.2296	28.9152	30.3768	30.5047
	−1	32.2685	32.3726	32.7820	32.9302	32.9569
	5	32.0104	32.0559	32.1552	32.0739	32.0916
	10	28.2675	29.4416	29.8102	30.1497	30.1912
Decenter error *δ*/μm	2.0	27.8829	29.3994	29.4021	30.6986	30.6988
	4.0	22.4982	26.5645	26.5748	28.9067	28.9081
	6.0	19.3433	25.7290	25.7774	28.9735	28.9756
	8.0	17.1759	27.2166	27.2793	30.4878	30.4928
	10.0	15.5782	27.5018	27.5799	31.9829	31.9908

**Table 3:** Correction results for distorted light-field images with different orientation errors.

Errors		Without Correction	Geometric Correction	Grayscale Correction
Coupled distance error Δ*z*/μm	−15	22.8892	29.7112	29.7426
	−10	25.9022	31.6473	31.6594
	−5	31.0463	34.2698	34.2597
	5	30.7221	34.1088	34.1179
	10	25.1907	31.9324	31.9624
	15	21.8477	30.3313	30.3676
Translation error Δ*t_x_*/μm	5	15.7850	31.3175	31.3192
	10	11.3673	31.1689	31.1711
	15	9.2537	31.0213	31.0235
Δ*t_y_*/μm	5	15.7861	31.3819	31.3837
	10	11.3678	31.0084	31.0105
	15	9.2537	31.0483	31.0503
Tilt error θ/°	0.2	25.6067	32.7456	32.7587
	0.4	20.3286	31.1000	31.1177
	0.6	17.5186	29.1576	29.1997
	0.8	15.8102	26.8395	26.9160
	1.0	14.6833	25.4762	25.6626

## References

[1] Ng R., Levoy M., Brédif M., Duval G., Horowitz M., Hanrahan P. (2005). Light field photography with a hand-held plenoptic camera. Stanford Tech. Rep. CTSR.

[2] Palmieri L., Scrofani G., Incardona N., Saavedra G., Martínez-Corral M., Koch R. (2019). Robust depth estimation for light field microscopy. Sensors.

[3] Cai Z., Liu X., Tang Q., Peng X., Gao B.Z. (2018). Light field 3D measurement using unfocused plenoptic cameras. Opt. Lett..

[4] Ma Y., Zhou W., Qian T., Cai X. A depth estimation algorithm of plenoptic camera for the measurement of particles. Proceedings of the IEEE International Conference on Imaging Systems and Techniques.

[5] Bae D.H., Kim J.W., Heo J.P. (2019). Content-aware focal plane selection and proposals for object tracking on plenoptic image sequences. Sensors.

[6] Xu C., Zhao W., Hu J., Zhang B., Wang S. (2017). Liquid lens-based optical sectioning tomography for three-dimensional flame temperature measurement. Fuel.

[7] Li T., Li S., Yuan Y., Wang F., Tan H. (2018). Light field imaging analysis of flame radiative properties based on Monte Carlo method. Int. J. Heat Mass Tran..

[8] Xu C., Zhang B., Hossain M.M., Wang S., Qi H., Tan H. (2016). Three-dimensional temperature field measurement of flame using a single light field camera. Opt. Express.

[9] Cao L., Zhang B., Li J., Song X., Tang Z., Xu C. (2019). Characteristics of tomographic reconstruction of light-field Tomo-PIV. Opt. Commun..

[10] Wu C., Ko J., Davis C.C. (2016). Imaging through strong turbulence with a light field approach. Opt. Express.

[11] Chen H., Woodward M.A., Burke D.T., Jeganathan V.S.E., Demirci H., Sick V. (2017). Human iris three-dimensional imaging at micron resolution by a micro-plenoptic camera. Biomed. Opt. Express.

[12] Song Y.M., Xie Y., Malyarchuk V., Xiao J., Jung I., Choi K.J., Liu Z., Park H., Lu C., Kim R.H. (2013). Digital cameras with designs inspired by the arthropod eye. Nature.

[13] Li Z., Xiao J. (2015). Mechanics and optics of stretchable elastomeric microlens array for artificial compound eye camera. J. Appl. Phys..

[14] Zhou P., Cai W., Yu Y., Zhang Y., Zhou G. (2018). A two-step calibration method of lenslet-based light field cameras. Opt. Lasers Eng..

[15] Li S., Zhang H., Yuan Y., Liu B., Tan H. (2016). Microlens assembly error analysis for light field camera based on Monte Carlo method. Opt. Commun..

[16] Li S., Yuan Y., Shen S., Tan H. (2019). Identification and correction of microlens array rotation error in plenoptic imaging systems. Opt. Lasers Eng..

[17] Li S., Yuan Y., Liu B., Wang F., Tan H. (2018). Influence of microlens array manufacturing errors on light-field imaging. Opt. Commun..

[18] Li S., Yuan Y., Liu B., Wang F., Tan H. (2018). Local error and its identification for microlens array in plenoptic camera. Opt. Lasers Eng..

[19] Shih K., Hsu C., Yang C., Chen H.H. Analysis of the effect of calibration error on light field super-resolution rendering. Proceedings of the IEEE International Conference on Acoustics, Speech and Signal Processing.

[20] Shi S., Ding J., New T.H., Liu Y., Zhang H. (2019). Volumetric calibration enhancements for single-camera light-field PIV. Exp. Fluids.

[21] Kong X., Chen Q., Wang J., Gu G., Wang P., Qian W., Ren K., Miao X. (2018). Inclinometer assembly error calibration and horizontal image correction in photoelectric measurement systems. Sensors.

[22] Hall M.E., Guildenbecher R.D., Thurow S.B. (2017). Uncertainty characterization of particle location from refocused plenoptic images. Opt. Express.

[23] Liu H., Wang Q., Cai W. (2019). Assessment of plenoptic imaging for reconstruction of 3D discrete and continuous luminous fields. J. Opt. Soc. Am. A.

[24] Suliga P., Wrona T. Microlens array calibration method for a light field camera. Proceedings of the 19th International Carpathian Control Conference.

[25] Su L., Yan Q., Cao J., Yuan Y. (2016). Calibrating the orientation between a microlens array and a sensor based on projective geometry. Opt. Lasers Eng..

[26] Bok Y., Jeon H., Kweon I.S. (2017). Geometric calibration of micro-lens-based light field cameras using line features. IEEE Trans. Pattern Anal. Mach. Intell..

[27] Cho D., Lee M., Kim S., Tai Y.W. Modeling the calibration pipeline of the lytro camera for high quality light-field image reconstruction. Proceedings of the IEEE International Conference on Computer Vision.

[28] Jin J., Cai W., Cao Y., Zheng W., Zhou P. An effective rectification method for lenselet-based plenoptic cameras. Proceedings of the Optoelectronic Imaging and Multimedia Technology IV.

[29] Li S., Zhu Y., Zhang C., Yuan Y., Tan H. (2018). Rectification of images distorted by microlens array errors in plenoptic cameras. Sensors.

[30] Liu B., Yuan Y., Li S., Shuai Y., Tan H. (2015). Simulation of light-field camera imaging based on ray splitting Monte Carlo method. Opt. Commun..

[31] Kim H.M., Kim M.S., Lee G.J., Yoo Y.J., Song Y.M. (2019). Large area fabrication of engineered microlens array with low sag height for light-field imaging. Opt. Express.

[32] Liu X., Zhang X., Fang F., Liu S. (2016). Identification and compensation of main machining errors on surface form accuracy in ultra-precision diamond turning. Int. J. Mach. Tool Manu..

[33] Zhang Q., Zhang C., Ling J., Wang Q., Yu J. (2018). A generic multi-projection-center model and calibration method for light field cameras. IEEE Trans. Pattern Anal. Mach. Intell..

[34] Wang Z. (2004). Image Quality Assessment: From Error Visibility to Structural Similarity. IEEE T. Image Process..

